# Assessment of Patients' Adherence to Antiepileptic Medications at Dessie Referral Hospital, Chronic Follow-Up, South Wollo, Amhara Region, North East Ethiopia

**DOI:** 10.1155/2018/5109615

**Published:** 2018-09-09

**Authors:** Gizachew Kassahun, Getachew Moges, Yitayew Demessie

**Affiliations:** ^1^Department of Pharmacognosy, School of Pharmacy, College of Medicine and Health Sciences, University of Gondar, P.O. Box 196, Gondar, Ethiopia; ^2^Department of Pharmaceutics and Pharmacoepidemiology, School of Pharmacy, College of Health Sciences, Wollo University, Dessie, Ethiopia; ^3^Department of Biomedical Sciences, College of Veterinary Medicine and Animal Sciences, University of Gondar, Gondar, Ethiopia

## Abstract

An epileptic seizure is a clinical event presumed to result from an abnormal and excessive neuronal discharge. The clinical symptoms are paroxysmal and may include impaired consciousness and motor, sensory, autonomic, or psychic events perceived by the subject or an observer. Epilepsy occurs when 2 or more epileptic seizures occur unprovoked by any immediately identifiable cause. And in the majority of patients with epilepsy, antiepileptic drugs effectively control their illness. However, more than 30% of people with epilepsy do not attain full seizure control, even with the best available treatment regimen. The aim of this study is to assess self-reported adherence in adult patients with epilepsy and to identify potential barriers for nonadherence to antiepileptic drug treatment in Dessie Referral Hospital. A hospital based cross-sectional study was conducted using structured questionnaires including Morisky medication adherence scale and analysis was conducted descriptively using SPSS version 20. The level of nonadherence to antiepileptic medication regimens was 34.1%. The major reason for missing medication was forgetfulness 53.5%. And the most common side effect was sedation 56.2%.* Conclusion.* Majority of epileptic patients in Dessie Referral Hospital was adherent to their AEDs treatment and among the determinants of adherence assessed the level of education and the side effect of drugs showed statistical significance.

## 1. Introduction

An epileptic seizure is a clinical event presumed to result from an abnormal and excessive neuronal discharge. The clinical symptoms are paroxysmal and may include impaired consciousness and motor, sensory, autonomic, or psychic events perceived by the subject or an observer. Epilepsy occurs when 2 or more epileptic seizures occur unprovoked by any immediately identifiable cause. The seizures must occur more than 24 hours apart. In epidemiologic studies, an episode of status epilepticus is considered a single seizure. Febrile seizures and neonatal seizures are excluded from this category [1].

Epileptic seizures are classified as cryptogenic or symptomatic. A cryptogenic seizure is a seizure of unknown etiology, and it is not associated with a previous central nervous system (CNS) insult known to increase the risk of developing epilepsy. A cryptogenic seizure does not conform to the criteria for the idiopathic or symptomatic categories [1].

In the majority of patients with epilepsy, antiepileptic drugs (AEDs) effectively control their illness. However, more than 30% of people with epilepsy do not attain full seizure control even with the best available treatment regimen [2]. One possible reason for treatment failure in epilepsy is poor adherence to AEDs. Nonadherence to AEDs has been found to be high [3]. For example, studies using insurance claims databases have reported that approximately 30–50% of patients with epilepsy do not adhere to their prescribed AED regimens [4]. Another study found that 70% of patients reported AED dose omissions [5].

Medication nonadherence can have adverse effects on clinical and economic outcomes [4]. It is well established that nonadherence to antiepileptic drugs (AEDs) may lead to a loss of seizure control [6].

Studies indicated that between 25% and 75% of people with epilepsy fail to follow prescribed drug regimens. This reduces the benefit that could be gained from antiepileptic drug leading to uncontrolled epilepsy and reduces quality of life [7]. A study conducted in Jimma University Specialized Hospital, South West Ethiopia, to determine adherence rate to antiepileptic medications and identify the potential risk factors associated with nonadherence indicated that, of a total of 265 patients studied, 63.2% of the patients were adherent. And the most common reason for missing dose was forgetfulness (31.8%) followed by being busy (20.9%). And sedation (39.4%) was the commonest side effect faced by the patient. And pill burden, comorbid conditions, and appointment missing were found to affect adherence [8].

A research was done in Nigeria, to assess patients' adherence to antiepileptic drugs. From 120 subjects information was obtained about patients' baseline characteristics, estimated monthly income, and determinants of adherence. Adherence to the AEDs was categorized as good, moderate, or poor. Good adherence was recorded in 70% of participants; 26.7% had moderate adherence and poor in remaining 3.3%. Education was statistically significantly associated with adherence. And the adherence rate among patients in this study was high despite poor control of seizure [9].

In a study carried out in UK Southampton General Hospital, to investigate nonadherence to antiepileptic drug treatment among patients with epilepsy in secondary care and the associations between adherence and seizure control, perceptions of illness and medication, anxiety and depression, of 54 patients, 59% were estimated to be nonadherent to medication. And there was a negative correlation between adherence and frequency of seizures and patients with poorly controlled epilepsy were more anxious and expected a longer duration of their epilepsy [6].

A study conducted in Asia, to assess rate of adherence in patients with epilepsy who missed their medications and reasons for nonadherence indicated that of a total of 368 patients studied 48.1% of patients were nonadherent with regard to AEDs. There were no demographic differences (based on gender, age, seizure type, and rural or urban location) between adherent and nonadherent patients. Adherence was positively and significantly correlated with duration of illness (*p*=0.007). The primary reason for nonadherence was forgetfulness or not having medication on hand (69.6%), followed by a negative attitude (12.8%), a bad patient-prescriber relationship (9.5%), side effects (5.4%), inability to buy drugs (1.9%), and other reasons (0.8%) [10].

## 2. Methods and Materials

### 2.1. Study Area and Study Period

This study was conducted from April to June 2017 in Dessie Referral Hospital, which is located 401 km North of Addis Ababa, Ethiopia, Amhara Region, South Wollo zone. Dessie Referral Hospital is a large institution serving Dessie town and the surrounding population of about 7 million.

### 2.2. Sample Size Determination and Sampling Procedure

Since the total epileptic patients in Dessie Referral Hospital were 112, all the patients were included in the study but patients who did not consent to participate and incomplete questionnaires were also excluded from the study. And some did not met the inclusion criteria. Patients above the age of 60 yrs were not included the study. All patients of either gender aged below 18 years, diagnosed with epilepsy and with no change in antiepileptic drug therapy in the last 3 months, attending the outpatient Departments of Medicine, Neurology, inpatients of medical wards, or those presenting to the emergency room of a tertiary care hospital were not recruited. This made the sample size to a convenience sample of** 88 **patients.

### 2.3. Data Collection and Management

To extract data, a structured interview questionnaire was administered to the study participants. The questioner contained sections that assessed sociodemographic, medication adherence, and questions related to antiepileptic drug use. The questionnaire was first prepared in English and translated into Amharic, the local language, and back translated to English to ensure that it retained its intended meaning. The questionnaire was pretested to identify potential problem and unanticipated interpretations, to any of the questions on 10 respondents having similar characteristics at the same facility to the study subjects on nonparticipants.

### 2.4. Data Entry, Analysis, and Interpretation

The data collected in the present study were entered to and analyzed using Statistical Packages for Social Sciences (SPSS) version 20. In the analysis, frequencies and percentages were used in the description of the data collected.

### 2.5. Ethical Considerations

Ethical approval was obtained from the ethical review committee of Department of Pharmacy, College of Medicine and Health Science, Wollo University, and letter of permission was obtained from the hospital neurologic clinic based on the request of the department. Besides, study participants were asked for informed oral consent and their information was maintained confidentially.

## 3. Results

From all epileptic patients (112) that have follow-up in DRH, only** 88 **patients (78.6%) consented and completed the interview.

### 3.1. Sociodemographic Variables

Out of 88 respondents, 55(62.5%) were males. Majority of the patients were in the age range of 18-38 (78.4%) and only 4 (4.8%) patients were above the age of 52.

Majority of the respondents 54 (61.4%) were Muslim by religion and half of the total patients were single. And about 51 (58%) of the patients have got access to primary education.

Around 20 (22.7%) of the respondents were student, 19 (21.6%) were daily labor, and 15 (17%) were government employee. And 26 (29.5%) of the patients have got an income in the ranges of 500-1000.

### 3.2. Side Effects Perceived by Patients

From the total sample size of 88 patients, 33 (37.5%) of them experienced side effect from their AEDs. And sedation was the most common side effect which was perceived by the patients 18 (56.2%). Details of the types of side effects experienced by the patients are presented in (Table 1).

### 3.3. Rate of Adherence to AED

Based on MMAS 58 (65.9%) of patients were classified as adherent.

### 3.4. Patients Missed Their Antiepileptic Medication

71 (80.7%) of patients missed the AED.

### 3.5. Reasons for Missing Medications

Forgetfulness was reported as a major reason by 38 (53.5%) of the patients and 18 (25.4%) of the patients miss their medication because of busy work schedules (Table 2).

### 3.6. Perception of Epileptic Patients about Their Illness

Around 34 (38.6%) of the respondents know that epilepsy is a neurological disease but some of the patients 26 (29.5%) had no idea of their illness and some 26 (29.5%) think that their illness is spiritual.

### 3.7. Duration on Antiepileptic Treatment

**Figure 1:**
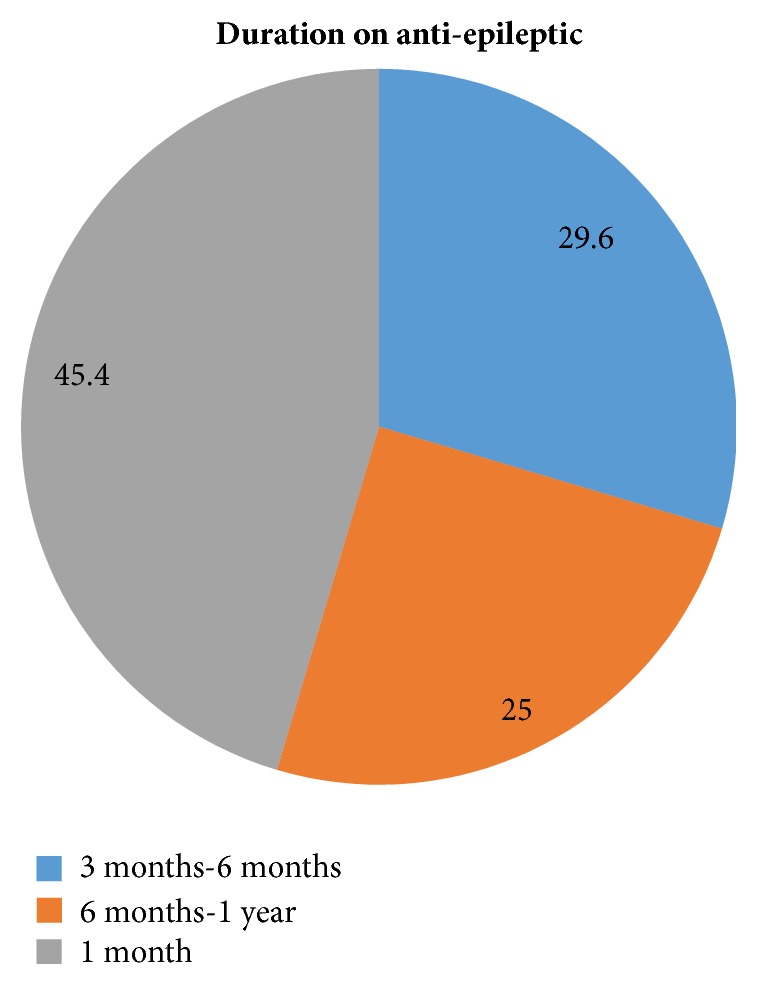
Patient's duration on AEDs at DRH, Dessie, Ethiopia, from April to June 2017.

40 (44.6%) of the patients were on AEDs for more than 1 year and 26 (31.3%) were started recently (6 month-1 year) (Figure 1).

### 3.8. Patients Taking Additional Medicine

Only 16 (18.2%) of patients took another medication in addition to their AEDs.

### 3.9. Epileptic Patients with Comorbid Condition

From 88 patients, only 12(13.6%) of them had any other chronic illness. And from those who had chronic illness 6 (50%) reported that it is heart failure, and 3 (25%) of them said that it is stroke (Table 3).

### 3.10. Test of Significance Using Pearson Chi Square

The Pearson chi square test was used to determine whether an association exists or not between the dependent variable (the level of patients adherence) and the independent variables (sex, monthly income, educational status, age, duration on AED treatment, comorbid conditions, and other medications taken with AEDs). Among determinants of adherence assessed in this study, level of education and side effect of drugs show statistical significance. Most side effects were associated with a significantly reduced likelihood of adherence (Table 4).

## 4. Discussion

This study showed that 34.1% of patients attending in Dessie Referral Hospital were nonadherent. And this finding was similar to a study done by Tefera Abula (30%) in Ethiopia [11], Dr Sunday O et al. (30%) in Legos [9], and Collin A et al. (29%) in Tennessee [12]. But some other studies reported a higher percentage of drug nonadherence, for instance, a study done in Nigeria by Ogboi S et al. reported 64.7 percentage of drug nonadherence [13]. This variation between results may be due to the difference in data collection method or due to the larger sample sizes used in the other studies.

Other studies showed that drug nonadherence among epilepsy patients ranges from 30% to 70% Buck D et al. [7]. Large differences in the adherence rate in different studies are due to different methods used for adherence assessment. Even with the same assessment tool, for instance, self-reported adherence, lack of validated questionnaires gives different adherence rate from different studies. These might be the reason why the findings of this survey differ from Walled M et al. [14] and from Jones RM et al. [6]. Both of them reported higher percentage of drug nonadherence compared to this study, even if they used the Morisky questionnaires to measure adherence.

Self-reported adherence was considered by many investigators as a good tool for adherence assessment but others reported that patients tend to overvalue their adherence [15]. This might be the reason why the percentage of drug adherence found in this study (66.3%) is higher when it is compared with a study done in Kenya that measures AEDS blood level [16].

Forgetfulness 53.5% was the primary reason for missing their medication which was followed by being busy 25.4% in this study. Other studies also exposed this factor: a study done in Asia by Tu, Luong Mac et al. revealed forgetfulness or not having medication on hand 69.6%, as the major reason for being nonadherent which was followed by a negative attitude 12.8% [17]. And a survey done by Tefera Abula also indicated that forgetfulness, lack of money, and side effects were the key reason for being noncompliance [11]. The reason why forgetfulness and being busy were the main reason to miss their medication might be most of the patients are on the working age so they may get busy at work and forget to take their medication.

In this study only 38.6% of the patients knew that epilepsy as a neurological disorder. But the others thought that as a spiritual disease (29.5%) and some they did not even have a clue about the disease (29.5%). A study done in Jimma by Getachew H et al. also revealed that 53.3% of patients had no idea about epilepsy and 18.8% thought that as a spiritual disease [8]. The reason why patients had no idea or did think it is a spiritual condition is might be because there is no way for them to know about the disease except a few moment conversations with their physician.

Among determinants of adherence assessed in this study, level of education and side effect of drugs show statistical significance. And this finding was similar to a study done in Nigeria by Dr Sunday O et al. [9]. The reason why educational status was associated with adherence might be explained by the fact that 58% of the patients in this study completed at least primary school and the reason why side effect of drugs associated with adherence might be 37.5% of patients experienced side effects.

The other sociodemographic variables assessed in this study like age, sex, and monthly income were not associated with adherence. This finding was also seen on a study done by Dr Sunday O et al. in Nigeria [9] and with a study done by Collin A et al. in Tennessee Health Science Center [12]. In this survey duration on AEDs, comorbid chronic illness and other medications taken with AEDs were not statistically associated with adherence.

## 5. Conclusion

In conclusion more than two-thirds of the study participants were found to be adherent to their AEDs treatment and forgetfulness which is followed by being busy which is the main reason for being nonadherent. In this study one-third of the patients had experienced side effects. And sedation was the commonest side effect faced by the patients. Among the determinants of adherence assessed, the level of education and side effect of drugs showed that statistical significance.

## Figures and Tables

**Table 1:** Perceived type of side effect experienced by patients due to AED regimen at DRH, Dessie, Ethiopia, from April to June 2017.

**type of side effect**	**Frequency**	**Percent (**%**)**
Rash	3	9.1
Sedation	18	54.5
Gingival hyperplasia	7	21.2

Cognitive impairment	4	12.1
Gastrointestinal discomfort	1	3.1
Total	33	100

**Table 2:** Patient's reason for missing their antiepileptic medication at DRH, Dessie, Ethiopia, from April to June 2017.

**Reason for missing medications**	**Frequency**	**Percent**
Forgetfulness	38	53.5
Being busy	18	25.4
Too many medication	2	2.8
Lack of hope on medications	2	2.8

Side effect	5	7.1
Cost	6	8.4
Total	71	100.0

**Table 3:** Epileptic patients with other chronic illness at DRH, Dessie, Ethiopia, from April to June 22 2017.

**Other diseases**	Frequency	Percent (%)
Heart failure	6	50
Stroke	3	25
Others	3	25
Total	12	100.0

Key: other RVI.

**Table 4:** Association of Adherence with possible determinants of adherence at DRH, Dessie, Ethiopia, from April to June 2015.

Variable	Adherent	Nonadherent	total	p-value
Sex				
Male	35	20	55	
female	23	10	33	0.561

Income				
<1000	32	26	58	
>1000	20	10	30	0.421

education				
illiterate	6	4	10	0.014
educated	52	26	78	

Age				
<52	54	30	84	0.623
>52	4	0	4	

duration				
<1 year	30	18	48	0.325
>1 year	28	12	40	

Other medicines				
Yes	11	5	16	0.791
No	47	25	72	

comorbid				
Yes	8	4	12	0.952
No	50	26	76	

Side effect				
Yes	26	7	33	0.048
No	32	23	55	

## Abbreviations

AEDs: Antiepileptic drugs
DRH: Dessie Referral Hospital
MMAS: Morisky medication adherence scale
RVI: Retroviral Infection.

## Additional Points

*Limitation. *Being a cross-sectional design conducted at a single university hospital, our study findings cannot be generalized to all areas of the country. Like most interview based researches, the study findings might be affected by the respondents' willingness to provide correct information and the way they understand interview questions. Moreover, the small sample size utilized in this study and the convenience sampling might also affect representativeness to all patients in the hospital.
